# Food supplements – the labelling challenges

**DOI:** 10.1177/02601060251388212

**Published:** 2025-10-28

**Authors:** Maria João Campos, Angelina Pena

**Affiliations:** LAQV-REQUIMTE, Laboratory of Bromatology, Pharmacognosy and Analytical Sciences, Faculty of Pharmacy, 37829 University of Coimbra, Coimbra, Portugal

**Keywords:** Food supplements, labelling, biotine, critical analysis, healthcare professionals

## Abstract

**Background:** Food supplements (FSs) labelling presents challenges for the food and pharmaceutical industries as well as regulatory authorities. Effective standardisation, improvement, and interpretation of FS labelling are essential for healthcare professionals (HPs) to provide accurate advice on these products. **Aim:** This article aims to address the regulatory and labelling complexities of these special foods, highlighting the need for more complete harmonisation all over the world and, at least, on the European Union (EU) market. **Methodology:** To help improve HP's knowledge concerning FS labelling, a critical analysis of relevant Global and European legislation and publications concerning FS labelling was conducted. **Findings:** It was found that FS labelling legislation is comprehensive. However, areas such as health claims and permitted dosages (illustrated by the case of biotin) require in-depth knowledge to develop guidelines to support HP interventions. The lack of harmonisation in EU regulation, for example, is a challenge that makes it difficult for HP to interpret information. The introduction of databases, such as the one developed by the Office of Dietary Supplements in the USA, which includes images of label quantities, serves as a model for providing transparent information. **Conclusion:** An HP with a better understanding of FS labelling is essential to provide accurate patient guidance and ensure the safety of FS consumption. The creation of resources and training programs to empower HPs is crucial to ensure quality advice and safe product use.

## Introduction

Food supplements (FSs) are distinct from conventional foods. They are designed to supplement, not replace, a regular and healthy diet. They provide concentrated sources of nutrients or other active substances that have nutritional or physiological effects. These products are available in pre-determined, measured doses, such as capsules, tablets, liquids, or powders (EFSA, 2025; THE EUROPEAN PARLIAMENT AND THE COUNCIL OF THE EUROPEAN UNION, 2002). A wide range of nutrients (micro and macronutrients) and other active ingredients might be present in FS, including vitamins, minerals, amino acids, essential fatty acids, fibre, and various plants and plant extracts, among several others (EFSA, 2025; THE COMMISSION OF THE EUROPEAN COMMUNITIES, 2006). FS is intended to correct nutritional deficiencies, maintain adequate intake (AI) of nutrients, or support specific physiological functions. They are not medicines and, as such, cannot exert a pharmacological, immunological, or metabolic action. Therefore, their use is neither intended to treat nor prevent human diseases nor to modify physiological functions (Ministério da Saúde, 2006; THE EUROPEAN PARLIAMENT AND THE COUNCIL OF THE EUROPEAN UNION, 2001).

There is no global agreement on how to categorise FS. Different countries all over the world use varying terminology and have different dosage requirements to classify a product as an FS or a medicine. This inconsistency creates complexity in international trade and regulation (Di Martino, 2025). The introduction to the market is also not harmonised. The regulatory framework in many countries mandates pre-market notification or registration for FSs before they can be legally commercialised (Di Martino, 2025). The Food and Agriculture Organisation (FAO) advocates for the global adoption of harmonised standards, such as the Codex Alimentarius, to simplify regulatory frameworks, ensure consistency, and facilitate international trade. This would help to standardise the rules for FSs on a global level (Di Martino, 2025).

In the European Union (EU), FS are regulated as food. Harmonised legislation regulates the vitamins and minerals and the substances used as their sources, which can be used to produce FS (THE COMMISSION OF THE EUROPEAN COMMUNITIES, 2009). The European Commission has defined harmonised rules to protect consumers against potential health risks associated with ingredients other than micronutrients (THE COMMISSION OF THE EUROPEAN COMMUNITIES, 2009).

Furthermore, FS cannot replace a regular and healthy diet, it only aims to increase human health and well-being (MINISTÉRIO DA AGRICULTURA E DO MAR, 2015). Legislation is a key factor in determining what kind of claims can be made on FSs and functional foods. It also dictates the level of scrutiny and oversight applied to those claims (Di Martino, 2025).

The labelling of FS varies considerably between countries around the world, although some standard requirements, such as the manufacturer's details and ingredient lists, are standard. In Africa, Nigeria, the labelling of FS is regulated by the Herbal Medicine and Related Products Labelling Regulations of 2019. Mandatory labelling, which must be in English, includes the brand name, ingredient list, net content, manufacturer's name and address, directions for safe use, lot number, expiration dates, and storage conditions (NAFDAC, 2019a, 2019b). In South Africa, health supplements must adhere to the labelling regulations outlined by the Medicines and Related Substances Act, 1965. Labels must include the nutrient reference value (NRV), an indication of artificial sweeteners, the name of the food, manufacturer details, instructions for use, net contents, country of origin, a list of ingredients, the batch identification number, and a statement emphasising that a varied diet is the most effective way to achieve good nutrition (SAHPRA, 2010). In Egypt, FS labels must include the product's name and description, ingredient list, net weight, producer/importer details, country of origin, batch number, expiration date, and nutritional facts. Mandatory warnings include not exceeding the recommended daily dose, that the product is not a substitute for a varied diet, and to keep it out of children's reach. Specific warnings for ingredients and against use by pregnant or lactating women are also required (NFSA, 2018). Similarly, in Australia, the labelling for complementary medicines has mandatory requirements for the product name, active ingredients, batch number, expiry date, warnings, storage conditions, and directions for use, all presented in English. General labelling standards also apply to all foods, covering identification, warnings, ingredient information, and nutritional claims (Food Standards Australia and New Zealand, no date).

Moving to Asia, in China, the Administrative Measures of Food Labelling govern the rules and regulations. Labels must include the food's name, the manufacturer's details, production and expiry dates, and the ingredient list. All information must be accurate and truthful (State Administration for Market Regulation, no date). Similarly, in India, the Food Safety and Standards (Labelling and Display) Regulations, 2020, prohibit claims that a product has the property of preventing, treating, or curing a human disease. Labels must include the product's category name (e.g. “health supplement”), the common name, the amount of nutrients, and warnings such as “not exceed the recommended daily usage,” “the product should be stored out of reach of children,” and “not for medical use” (FSSAI, 2020).

Across America, in Argentina, the labelling of FS, or “suplemento dietario,” must adhere to general food labelling regulations and specific requirements in Article 1381 of the CAA. Key elements include the legal designation “suplemento dietario,” brand name, net weight, a complete ingredient list, allergens, manufacturer details, country of origin, instructions for use, daily recommended intake, mandatory warnings, and the use by date (Ministerio de Salud, no date). In Brazil, specific labelling provisions are outlined in RDC No.243/2018 and Normative Instruction No.28/2018. Labels must include the name “Suplemento Alimentar,” instructions for use and storage, and warnings like “This product is not a medicine,” “Keep out of the reach of children,” and “Do not exceed the recommended daily intake.” However, the labelling of FSs must not include any representation, even in other languages, that explicitly or implicitly asserts, suggests, or insinuates that 1) the product serves medicinal or therapeutic purposes, 2) contains substances that are unauthorised or prohibited, 3) the food is incapable of supplying the necessary components for health, or 4) that the product is equivalent to or superior to traditional foods (ANVISA, 2018a, 2018b). In Canada, the general labelling requirements for Natural Health Products stipulate that the label must contain a principal display panel with the brand name, product number, dosage form, and net amount. The label should also show the name and address of the manufacturer, an ingredient list, recommended use, and risk information, including cautions and warnings (Health Canada, 2022). The United States, under FDA regulations, requires dietary supplement labels to bear five statements: the product name, the manufacturer's details, a “Supplement Facts” panel, a list of “other ingredients,” and the net quantity. The “Supplement Facts” panel must list the serving size and each dietary ingredient (US FDA, no date).

Finally, in Europe, the labelling of foods with added vitamins and minerals must not imply that a balanced and varied diet is inadequate, as said. The Directive 2002/46/EC specifies that FS labels must contain a cautionary warning against exceeding the recommended dose, a statement that they are not a substitute for a varied diet, and advice to keep them out of reach of young children. The directive also explicitly prohibits labelling that suggests the product can prevent, treat, or cure diseases (THE EUROPEAN PARLIAMENT AND THE COUNCIL OF THE EUROPEAN UNION, 2002).

While the labelling regulations for FS vary from country to country, the global trend points towards greater clarity, transparency, and consumer safety, with most regions requiring detailed information on ingredients, usage instructions, and safety warnings; however, harmonisation is needed. With this reflection, we aim to contribute to the creation of more harmonised labelling rules and to working tools and databases for dietary supplement labelling, which will be essential for a more robust understanding of these products, especially among healthcare professionals (HPs).

## Food supplementś labelling challenges

The regulation of FSs in the EU is not fully harmonised, creating significant challenges for HPs who advise patients on the use of FS. This lack of consistent legislation and the unclear distinction between FSs and medicines can lead to a product being classified as a medicine in one European country but an FS in another. This is possible because food legislation does not prohibit the inclusion of substances with pharmacological effects in FSs, such as plants and plant extracts. This regulatory inconsistency makes it difficult for professionals to access and interpret the necessary information about these products. To address this, creating resources to empower HPs is essential for providing higher quality advice to patients. For example, a comprehensive database of FSs, their labels, and ingredient lists would be beneficial for both HPs and consumers. The absence of standardised labelling practices across the world, particularly in the EU states, can potentially increase the risk of inappropriate FS use, highlighting the need for accessible and transparent information and consistent labelling standards. In the United States, the Office of Dietary Supplements at the National Institutes of Health developed the Dietary Supplement Label Database, which provides detailed product information, including images of package labels, ingredients, and all label statements (National Institutes of Health, no date). The wide range of locations where FSs are sold, from pharmacies to supermarkets, online or any other shops permitted to sell food, including minimarkets or car service stations, further complicates this issue by reducing the availability of clinical guidance.

Even for HPs, accessing reliable information on FS labelling is a twofold challenge: readily accessible resources detailing FS composition and labelling are often lacking, and even when available, the complexity of the information requires specialised knowledge for accurate interpretation. Effective training programs are crucial to equip HPs with the skills to understand FS labels and critically evaluate health claims. While Regulation 1169/2011 (THE EUROPEAN PARLIAMENT AND THE COUNCIL OF THE EUROPEAN UNION, 2011), provides a comprehensive framework for general food labelling, and European Commission Directive 2002/46/EC outlines specific mandatory requirements for FS labelling (THE EUROPEAN PARLIAMENT AND THE COUNCIL OF THE EUROPEAN UNION, 2002).

A key element of FS labelling is the use of health claims, which are statements suggesting a relationship between a food and health, which is particularly relevant for HPs. The European Commission authorises these claims based on scientific evidence evaluated by the EFSA. A publicly accessible database allows for the verification of the 261 currently authorised health claims. Nutritional claims, while also present, are of less direct relevance to FS (European Commission Food and Feed Information Portal Database, no date a). Most health claims for micronutrients in Europe require the food to be at least a “source of” that nutrient, as listed in the Annexe to EC Regulation 1924/2006. As a condition of use, a food must meet a minimum of 15% of the NRVs per day (European Commission Food and Feed Information Portal Database, no date a; THE EUROPEAN PARLIAMENT AND THE COUNCIL OF THE EUROPEAN UNION, 2006).

However, interpreting the nutritional information on FSs can be confusing because many products have vitamin and mineral values far exceeding the 15% NRV threshold, and even much higher than 100%. To address this, it is essential to understand both NRV and AI values. The EFSA's Dietary Reference Value (DRV) Finder is a valuable online tool that provides easy access to DRVs, which are science-based NRVs for healthy populations. It is essential to understand that DRVs are scientific benchmarks for professionals to formulate recommendations for groups, not individual recommended intakes (EFSA, no date). HPs should adjust these values to fit the nutritional needs of people with specific clinical conditions or diseases (THE EUROPEAN PARLIAMENT AND THE COUNCIL OF THE EUROPEAN UNION, 2006).

The EFSA's DRV Finder is a valuable online tool that provides easy access to DRVs for nutrients. Its resource is designed for a wide range of users, including HP, risk managers, policymakers, food manufacturers, and researchers. DRVs are science-based NRVs established for healthy populations and are specific to different life stages and genders. These values serve multiple purposes, such as evaluating the nutritional adequacy of diets for individuals and groups, informing the design of dietary plans (e.g. school meals), guiding the development of nutrition guidelines, supporting dietary counselling, contributing to the establishment of food labelling reference values, and shaping nutrition and food policies. However, it is essential to understand the scientific basis behind DRVs, as well as the difference between their application to individuals versus groups, for proper interpretation. DRVs should not be seen as recommended intakes for individuals but rather as scientific benchmarks for professionals to develop personalised recommendations. Policymakers also use DRVs when setting reference values for food labelling. EFSA's role is limited to establishing the DRVs, and determining nutrition goals, guidelines, or labelling requirements falls outside their remit. Finally, DRVs are intended for healthy individuals, and HP should adjust these values to meet the nutritional needs of individuals with specific clinical conditions or illnesses (EFSA, no date; EFSA Panel on Dietetic Products, N. and A. (NDA), 2010).

## The specific case of biotin

Some individuals have increased nutritional needs due to unique characteristics or clinical conditions, leading market operators to create FS formulations with micronutrient values far exceeding the NRV. As an example, some FSs contain biotin with an NRV of 10,000% or more. Biotin is a crucial cofactor for key metabolic pathways, and its deficiency, while uncommon, can manifest in various symptoms. Chronic alcohol consumption, smoking, and certain medical conditions increase the risk of biotin deficiency. Biotin toxicity is rare, so no Tolerable Upper Intake Level has been established (Chang and Lipner, 2020). However, the FDA has issued warnings that biotin can interfere with laboratory tests (McSeveney, no date).

Biotin is a cofactor for enzymes involved in critical metabolic pathways, including gluconeogenesis, lipid metabolism, and amino acid catabolism. These metabolic functions are the basis for the authorised health claims for biotin, as summarised in Table 1 (European Commission Food and Feed Information Portal Database, no date a; European Commission Health Claims, no date b). In addition to its role in metabolism, adequate biotin intake is vital for healthy foetal development. Biotin is available in many of foods, making deficiency uncommon in the population. However, it can lead to dermal issues and neurological complications. Certain clinical conditions and lifestyle factors, such as chronic alcohol consumption, smoking, the use of anticonvulsant medications, and inflammatory bowel disease, can increase the risk of deficiency due to difficulty in biotin absorption or accelerated catabolism. Severe biotin deficiency can also be caused by genetic flaws in biotinidase and holocarboxylase synthetase, with symptoms from hallucinations and depression to seizures and developmental delays (Perry and Butterick, 2024). Furthermore, long-term antibiotic use may disrupt the gut microbiome, potentially reducing bacterial biotin production. Importantly, biotin toxicity is rare, and no Tolerable Upper Intake Level has been established (Berger et al., 2024).

**Table 1.** Health claims for biotin (European Commission Food and Feed Information Portal Database, no date a; European Commission Health Claims, no date b).

Health claims	Type of health claim	Conditions of use
Biotin contributes to normal energy-yielding metabolism.	*Health relationship:* energy-yielding metabolism.	The claim may be used only for food which is at least a source of biotin as referred to in the claim SOURCE OF [NAME OF VITAMIN/S]AND/OR [NAME OF MINERAL/S] as listed in the Annex to Regulation (EC) No 1924/2006.
Biotin contributes to the normal functioning of the nervous system.	*Health relationship:* function of the nervous system.	
Biotin contributes to normal macronutrient metabolism.	*Health relationship:* contribution to normal macronutrient metabolism.	
Biotin contributes to normal psychological function.	*Health relationship:* contribution to normal psychological functions.	
Biotin contributes to the maintenance of normal hair.	*Health relationship:* maintenance of normal hair.	
Biotin contributes to the maintenance of normal mucous membranes.	*Health relationship:* maintenance of normal skin and mucous membranes.	
Biotin contributes to the maintenance of normal skin.	*Health relationship:* maintenance of normal skin and mucous membranes.	

Assessing biotin status presents a challenge. While a clinical evaluation of suggestive symptoms – such as dermatitis, alopecia, or neurological manifestations – combined with a history of inadequate dietary intake can raise suspicion of a deficiency (Berger et al., 2024), traditional methods of measuring biotin levels in blood and urine may not accurately reflect a person's overall status. Although decreased urinary biotin excretion and increased urinary 3-hydroxyisovaleric acid excretion can be early and sensitive indicators of altered biotin status, these methods are not routinely used in clinical practice (Perry and Butterick, 2024). Current recommendations suggest that biotin status should be assessed through direct measurement of biotin in blood and urine, supplemented by the determination of biotinidase activity (Berger et al., 2024). A clinical evaluation is necessary to determine when and how to provide additional amounts. Reference values are summarised in Table 2 (THE EUROPEAN PARLIAMENT AND THE COUNCIL OF THE EUROPEAN UNION, 2011); EFSA, no date; Berger et al., 2024). Supplementation is strongly recommended for breastfeeding mothers, with an intake of at least 35 µg of biotin per day orally (Berger et al., 2024) or even 45 µg (EFSA, no date). Additional amounts may also be needed in patients on renal replacement therapy (Berger et al., 2024).

**Table 2.** Nutritional and dietary values of interest for biotin.

Type of value	Dosage	Reference
Nutrient reference values (NRVs)	50 µg	THE EUROPEAN PARLIAMENT AND THE COUNCIL OF THE EUROPEAN UNION, 2011)
AI (adults, including pregnant women)	40 µg	(EFSA, no date)
AI breastfeeding mothers	45 µg	(EFSA, no date)
Upper level	Not defined	(EFSA, no date)
DRI per day age 31–70 years	30 µg (AI)	(Berger et al., 2024)

AI: adequate intake.

This example highlights the necessity of accessible resources for researching FS ingredients and a thorough understanding of the meaning behind labelled values. Recommendations for FSs should ideally come from HPs who have detailed knowledge of ingredient effects, potential drug interactions, and the risks associated with excessive or concurrent intake.

This work has examined key aspects of FS labelling to enhance professional practice. We believe that to improve guidance on FS labelling in Europe, specific training programs to empower HPs to accurately interpret complex labels and provide safe, informed advice should be developed. A comprehensive, publicly accessible European database, similar to the U.S. model, should also be created to centralise detailed information on FS ingredients. This would help reduce consumer confusion and ensure that HP recommendations are based on verified data. Further research is warranted to increase awareness and promote FS's safe and effective use in terms of consumption and professional guidance.
